# A Bibliometric and Visualization Analysis of Motor Learning in Preschoolers and Children over the Last 15 Years

**DOI:** 10.3390/healthcare10081415

**Published:** 2022-07-28

**Authors:** Fei Xu, Jing Xu, Daliang Zhou, Hao Xie, Xuan Liu

**Affiliations:** 1 School of Physical Education, Hangzhou Normal University, Hangzhou 311121, China; feixu@hznu.edu.cn; 2 School of Physical Education, Nanjing Xiaozhuang University, Nanjing 211171, China; xujing3404@gmail.com (J.X.); p2007032@njxzc.edu.cn (D.Z.); 3 Department of Physical Education, Zhejiang University of Finance & Economics, Hangzhou 310018, China; miki.xie@gmail.com; 4 Division of Library and Information Services, Hangzhou Normal University, Hangzhou 311121, China

**Keywords:** motor learning, motor control, motor development, preschooler, children, knowledge domain visualization, hotspots, emerging trend, CiteSpace

## Abstract

Motor learning enables preschoolers and children to acquire fundamental skills that are critical to their development. The current study sought to conduct a bibliometric and visualization analysis to provide a comprehensive overview of motor-learning progress in preschoolers and children over the previous 15 years. The number of studies is constantly growing, with the United States and Australia, as well as other productive institutions and authors, at the leading edge. The dominant disciplines were Neurosciences and Neurology, Psychology, Rehabilitation, and Sport Sciences. The journals *Developmental Medicine & Child Neurology*, *Human Movement Science*, *Physical Therapy*, *Neuropsychology*, *Journal of Motor Behavior*, and *Journal of Experimental Child Psychology* have been the most productive and influential in this regard. The most common co-citations for clinical symptoms were for cerebral palsy, developmental coordination disorder, and autism. Research has focused on language impairment (speech disorders, explicit learning, and instructor-control feedback), as well as effective intervention strategies. Advances in brain mechanisms and diagnostic indicators, as well as new intervention and rehabilitation technologies (virtual reality, transcranial magnetic stimulation, and transcranial direct current stimulation), have shifted research frontiers and progress. The cognitive process is critical in intervention, rehabilitation, and new technology implementation and should not be overlooked. Overall, our broad overview identifies three major areas: brain mechanism research, clinical practice (intervention and rehabilitation), and new technology application.

## 1. Introduction

Motor learning is a collection of processes that result in relatively permanent changes in movement capability because of practice or experience [1]. It is a neural and cognitive process that occurs throughout human development and is deduced from skilled performance [2,3]. Skilled performance is a physical activity that is defined by parameters such as task completion time, accuracy, and movement coordination (efficiency, quality, and time) [1]. There is, however, more to learn than simply how to perform better. There is the practice-based development of the underlying capability for skilled performance, with better capability leading to better performance.

Motor learning enables children to acquire fundamental skills that are critical to their development [3]. However, difficulties acquiring movement skills are one of the most common developmental issues, affecting approximately 6% of all children [4,5]. These motor difficulties, whether or not related to a known medical condition, are more evident in early childhood, primarily involving developmental coordination disorder (DCD), cerebral palsy (CP), autism spectrum disorder (ASD), and attention deficit hyperactivity disorder (ADHD), are a risk factor for concomitant psychosocial and behavioral functioning problems [5,6]. For decades, researchers have conducted numerous studies at various levels in an attempt to investigate the clinical features and implement effective interventions and rehabilitation. Early research focused on the challenges and performance of children with motor-skill-learning deficits, such as internal modeling, motor control, and rhythmic coordination [7,8,9,10]. Children with ADHD and ASD had difficulty with basic motor control and expressing and recognizing skilled motor postures [11]. Some motor-deficit symptoms, on the other hand, appear early in development and are frequently identified at school age, leading to missed opportunities for early intervention [12]. It has been demonstrated that intrinsic motivation, learning attention [13], practice [14], feedback [14], and instruction [15] all help to improve motor skills and performance. Furthermore, new research indicates that combining motor imagery with physical exercises benefits children’s health and motor skills [16]. Overall, newborns, toddlers, and children have been shown to be at high risk of a variety of motor impairments due to a combination of biological and environmental risk factors [17]. Indeed, motor impairment is associated with comorbidities, which may have a greater impact on quality of life, academic achievement, and participation in extracurricular activities than the motor impairment itself [17].

Despite decades of research focusing on factors related to motor learning, the underlying mechanisms and how many cognitive factors contribute to motor-learning difficulties remain unknown. Researchers have focused their attention on children with neurological impairments who struggle to learn motor skills, whilst motor learning’s neural and cognitive processes are not obvious and must be deduced from changes in skilled performance [2]. It is widely accepted that motor learning is important for education and rehabilitation, particularly for children with CP [18], DCD [19], and ASD [11,20,21,22]. There are, however, no studies that summarize the hotspots and trends in this research topic. As a result, by summarizing the recent findings, we hope to advance this research topic. According to big-data bibliometric analysis, knowledge visualization is an important aspect of scientific investigation for evolution of a new research topic. CiteSpace, a knowledge-visualization software, is used in this scenario for comparative and bibliometric analysis [23,24]. Based on the literature, statistical analysis and visualization results were generated and presented to reflect the evolution of this research topic [23,24,25]. Thus, the current study sought to identify hotspots and emerging trends in the motor learning of preschoolers and children through bibliometric analysis using CiteSpace software.

## 2. Materials and Methods

### 2.1. Data Source and Search Strategy

The data for this analysis came from the Web of Science (WoS), the most widely used and trusted database of research publications and citations. The WoS includes multiple retrieval methods and retrieval conditions to fulfill the objectives. Science Citation Index Expanded (SCIE), Social Sciences Citation Index (SSCI), Arts & Humanities Citation Index (A&HCI), Emerging Sources Citation Index (ESCI), Current Chemical Reactions-Expanded (CCR-Expanded), and Index Chemicus (IC) are among the high-quality and impact journals available through Web of Science Core Collection (WoSCC). Therefore, it is preferable to investigate the relevant, complete, and accurate literature data using WoSCC. Briefly, the data-search strategy was as follows: (1) Topic = (child * OR preschool * OR kid * OR pupil OR kindergarten OR early child* OR young child *) NOT Topic = (adult OR adolescent OR teenager) AND Topic = (“motor learning”) NOT Topic = (mouse OR mice OR rat); (2) document type = article or review; (3) language = English; (4) timespan = 1975–2022 (see Supplementary S1). All data were obtained on 22 February 2022.

### 2.2. Data Analysis and Visualization Tools

We followed the search strategy and obtained legitimate data in strict accordance with the inclusion criteria. For further analysis, full records and cited references were downloaded in plain text format [26]. Duplicate and irrelevant studies were removed by two researchers. CiteSpace V5.8.R3 (64-bit) was used to display and analyze the research scene and progress. CiteSpace is a novel bibliometric analysis tool that combines data visualization with Java data-mining algorithms [23,24,26].

After the preceding steps were correctly completed and reconfirmed, we created two folders for CiteSpace analysis [26]. One contains WoSCC download data, while the other is a project folder. The search results revealed that the earliest study that met the inclusion criteria was in 2006. Therefore, the overall time span of our study was normalized as January 2006 to February 2022. Publication, countries/regions, institutions, journals, categories, references, and keywords are important features. The pathfinder function was allotted a link-reduction algorithm to provide a more reasonable and precise network configuration. Different nodes on a map represented indices such as a country, institution, or journal. The size of nodes indicated the frequency and centrality of publications, and a large node typically indicated a pivotal point with a high occurrence or citations. The node links represented a network of cooperation, collaboration, or co-citation, and the color of the nodes and links indicated different clusters. According to Kleinberg’s algorithm, an occurrence burst is a term that occurs frequently over a given period and can be regarded as research hotspots and frontiers [23,24].

## 3. Results

### 3.1. General Data

**Figure 1:**
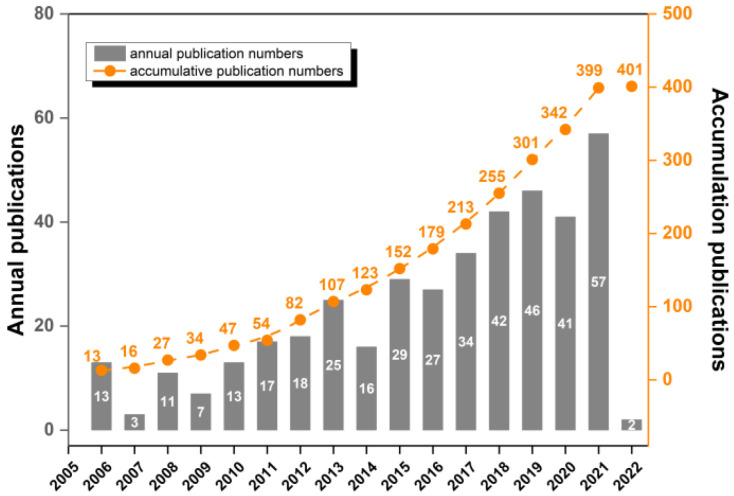
Annual and cumulative motor-learning publications for preschoolers and children.

After removing the redundant and irrelevant studies, 401 studies remained. They were all published in English between 2006 and 2022. The WoSCC conducted the first study in 2006 [7], based on clear results from the literature. Figure 1 depicts the number of studies (annual and cumulative publications, respectively), which has been steadily increasing from year to year over the last 15 years. Notably, the number of studies between 2015–2021 has increased significantly compared with the number of studies between 2005–2014.

### 3.2. Quantitative and Cooperation of Countries/Regions, Institutes, and Publications

**Figure 2:**
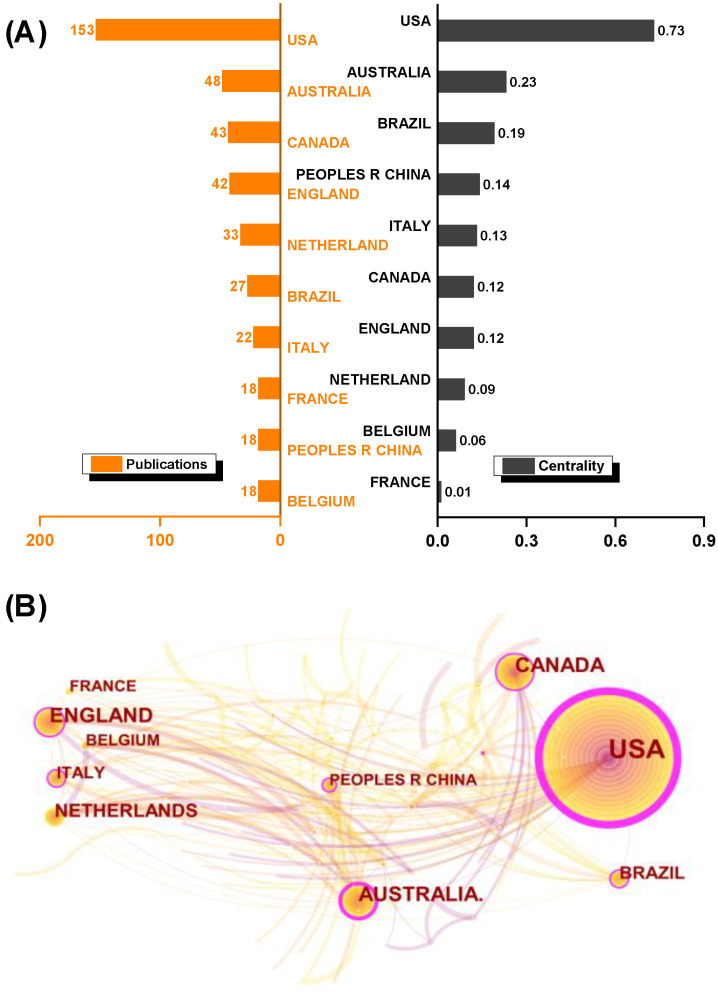
Top 10 productive countries/regions and their cooperation. (**A**) Publications and centrality of the top 10 productive countries/regions; (**B**) cooperation visualization. Note: the number of publications and centrality are represented by the size of the node ring and the thickness of the purple ring surrounding the node. The number and thickness of connecting lines represent the cooperative network [23,24].

Figure 2 displays the publications, centrality of countries/regions, and cooperation networks. Obviously, the United States has the largest node and the thickest purple ring. It has the most publications (153) and the highest centrality (0.73). Australia, Canada, the United Kingdom, and the Netherlands trail the United States in terms of publications. In terms of centrality, the United States is followed by Australia, Brazil, China, and Canada.

The cooperation-network visualization reveals that the top countries/regions in terms of publication and centrality have stronger connections with each other. France (ranked eighth in publications) and China (ranked ninth in centrality) emerge as having few links with other countries/regions. China is somewhat separated, despite its high centrality (ranked fourth), with fewer collaborative and links with other countries/regions.

**Figure 3:**
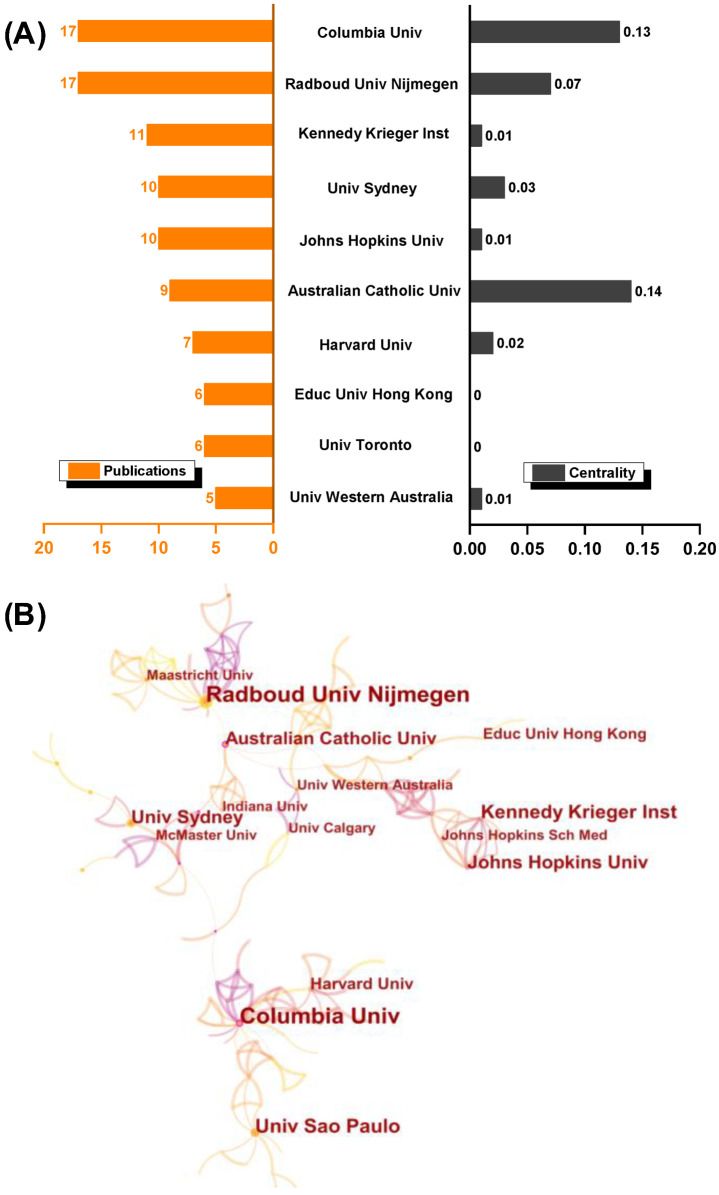
Top 10 productive institutions and their cooperative relations map. (**A**) Publications and centrality of top 10 institutions; (**B**) cooperative relations map of institutions.

The most productive institutions, according to Figure 3, are Columbia University (frequency 17) and Radboud University Nijmegen (frequency 17). The Kennedy-Krieger Institute (11), the University of Sydney (10), and Johns Hopkins University (10) are three of the latter. In terms of centrality, Australian Catholic University (0.14), Columbia University (0.13), and Radboud University Nijmegen (0.07) ranked first, second, and third, respectively. The network of institutional cooperation may be assumed to be weakly linked. It appears to be a dispersed “nebula” of small groups, with two or three well-known institutions collaborating closely.

### 3.3. Authors and Co-Cited Authors’ Network

**Figure 4:**
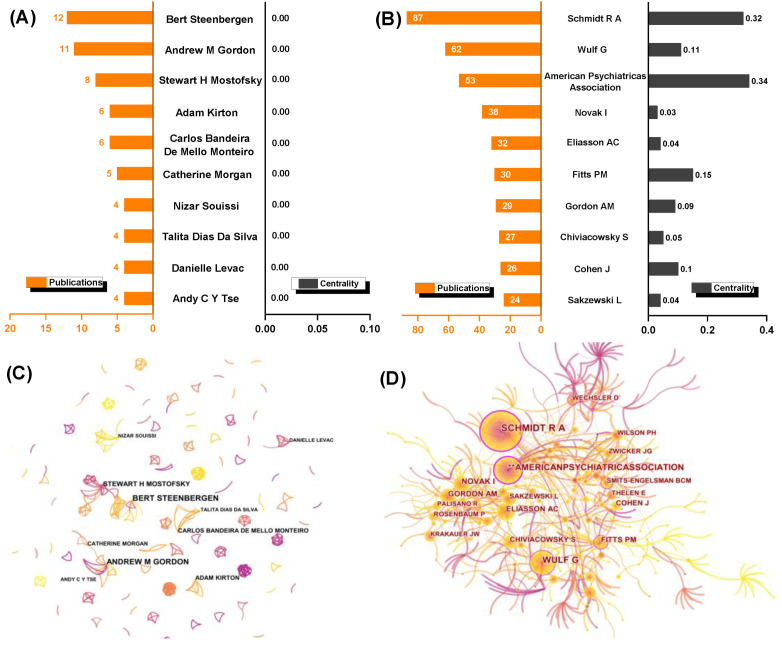
Contribution of authors and co-cited authors’ network in visualization. (**A**) Productive authors and (**B**) the frequency and centrality of co-cited authors; (**C**) contribution of authors and (**D**) the co-cited authors’ network map.

The most productive researchers in terms of current publications and contributions have been Steenbergen (Radboud University, Nijmegen, The Netherlands), Gordon (Columbia University, NY, USA), and Mostofsky (Kennedy Krieger Institute, Baltimore, Maryland, USA). Surprisingly, none of the top 10 authors are assigned a high centrality (Figure 4A). This is also graphically reflected in the contributing-author map. The productive authors appear to be leading their respective research teams in separation, with little collaboration and connection to other teams. The contributing-author map clearly illustrates this, with scattered nodes indicating that author cooperation is low (Figure 4C). The results for the co-cited authors, on the other hand, are entirely positive. Schmidt, Wulf, Novak, Fitts, Cohen, and other prominent researchers are among those with a high co-citation frequency and centrality (Figure 4B). The collaboration of highly cited authors reveals the recognition and acknowledged influence of the researchers who have made significant contributions to the field (Figure 4D).

### 3.4. Authors and Co-Cited Authors’ Network

**Figure 5:**
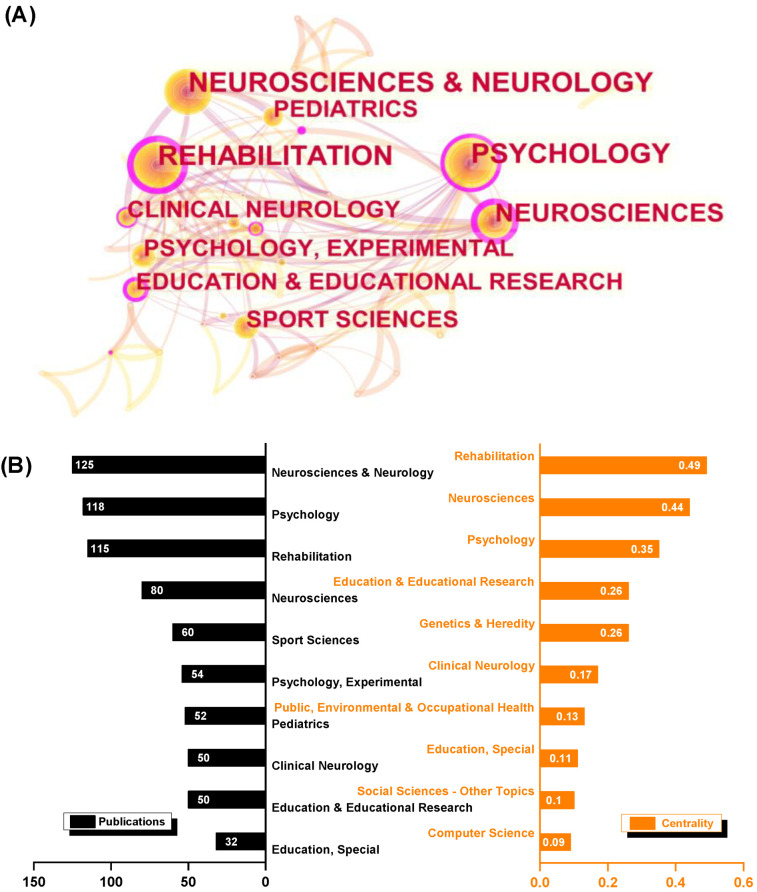
Research categories in visualization. (**A**) The categories’ network map; (**B**) top 10 categories in terms of publications and centrality.

Many categories have contributed to this topic, as shown in Figure 5A. Neurosciences and Neurology (125 publications)/Neurosciences (80), Psychology (118), Rehabilitation (115), and Sport Sciences are the most important categories (60). Furthermore, Rehabilitation (0.49) has the highest centrality, followed by Neurosciences (0.44) and Psychology (0.35).

### 3.5. Co-Cited and Top-10 Cited Journals

**Figure 6:**
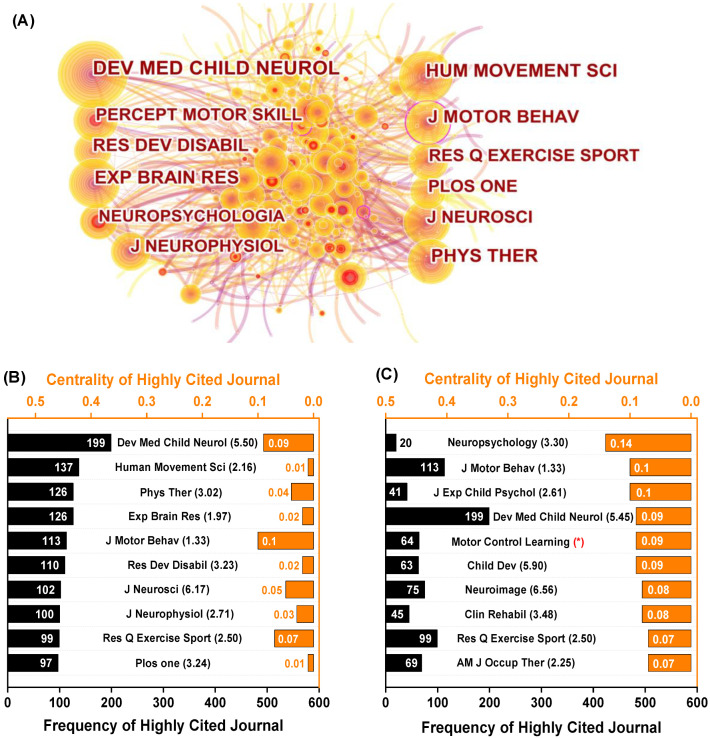
Visualization of co-cited and top-10 cited journals on preschoolers’ and children’s motor learning. (**A**) A collaborative map of highly cited journal references; and the top 10 journals in terms of frequency (**B**) and centrality (**C**). Note: the number in parentheses indicates the journal’s impact factor in 2021. * denotes a book rather than a journal.

The CiteSpace analysis included 430 journals, with a total of 6525 co-citations. Figure 6 illustrates information from the top 10 journals. The largest node is *Dev Med Child Neurol* (frequency: 199, centrality: 0.09), followed by *Hum Movement SCI* (137, 0.01), *Phys They* (126, 0.04), and *Exp Brain Res* (126, 0.02). Surprisingly, *Neuropsychology* has the lowest frequency (20) but the highest centrality (0.14). *J Motor Behav* (0.1), *J Exp Child Psychol* (2.61) and *Dev Med Child Neurol* (0.09) were ranked second to fourth in terms of centrality, respectively.

### 3.6. Co-Cited References

**Figure 7:**
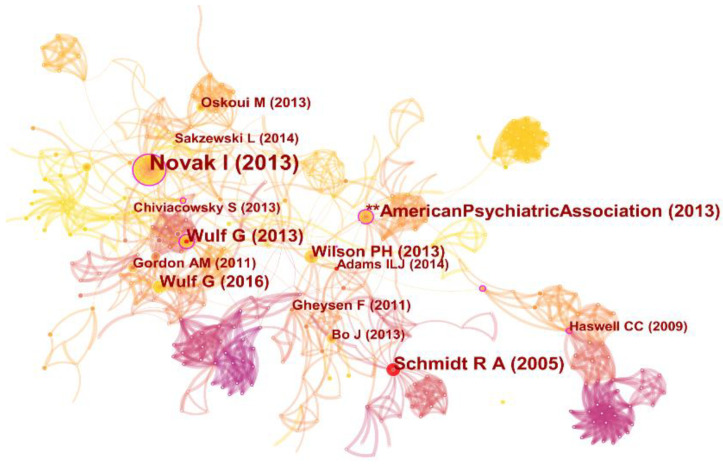
Visualization of co-cited authors. Note: the clusters are named by extracting nominal terms as labels from the titles of cited references. ** indicates a manual.

As the node type for analysis, 401 original records and 15,689 references were chosen. The three studies published by Novak [18], Schmidt [1], and the American Psychiatric Association [6] ranked highest in terms of co-citation frequency and centrality (Table 1). Another high-centrality meta-analysis [10] is also crucial. Notably, during the years of citation from 2006 to 2022, the visualization of the collaborative network of co-cited authors is stunning (Figure 7). The colors in the visualization map progress from pink to purple to orange. Novak (2013) has the biggest node with a purple ring, Schmidt has a red ring, and Wulf appears twice. To summarize, it is worth noting that the collaborative network of authors with high publications (Figure 4C) and high co-citations demonstrates two levels of linkage closeness.

### 3.7. Co-Cited References Cluster and Timeline

**Figure 8:**
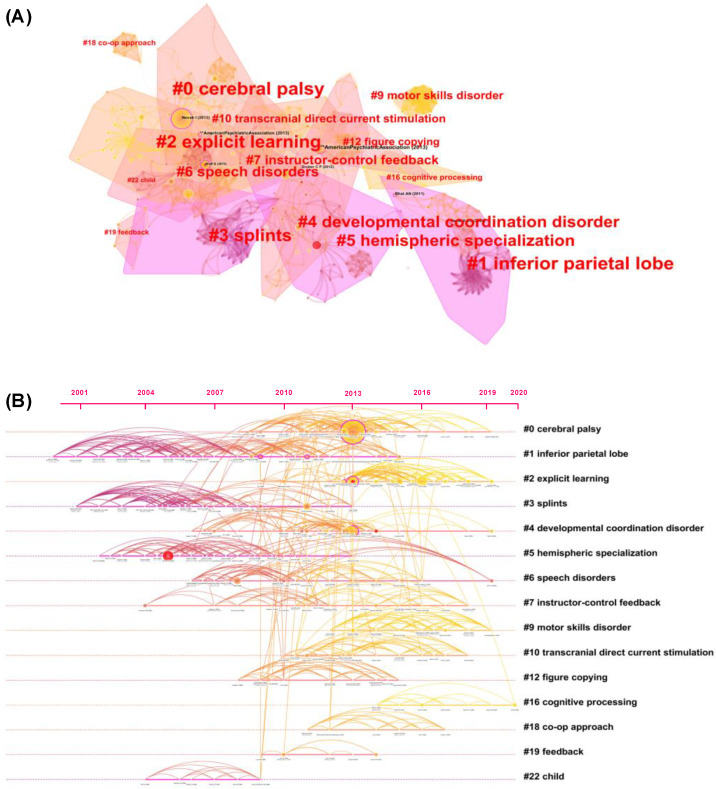
Visualization of co-cited references and a timeline cluster. (**A**) Cluster map of co-cited references; (**B**) timeline map of co-cited references. Note: silhouette > 0.7, the cluster is convincing [23,24].

Figure 8 depicts the network clustering and timeline visualization (the top 10 clusters are described in Appendix A). The clustering of co-cited references appears to be very blocky and indicator-based. Clustering results in preschoolers and children focused primarily on clinical symptoms (#0 CP, #4 DCD, #9 motor-skills disorder) and diagnostic indicators (#1 inferior parietal lobe, #5 hemispheric specialization). Language impairment (#6 speech disorders), education-based interventions (#2 explicit learning, #7 instructor-control feedback), and new intervention strategies (#10 transcranial direct-current stimulation, tDCS) are also noteworthy.

### 3.8. Keywords and Keywords Clusters

**Figure 9:**
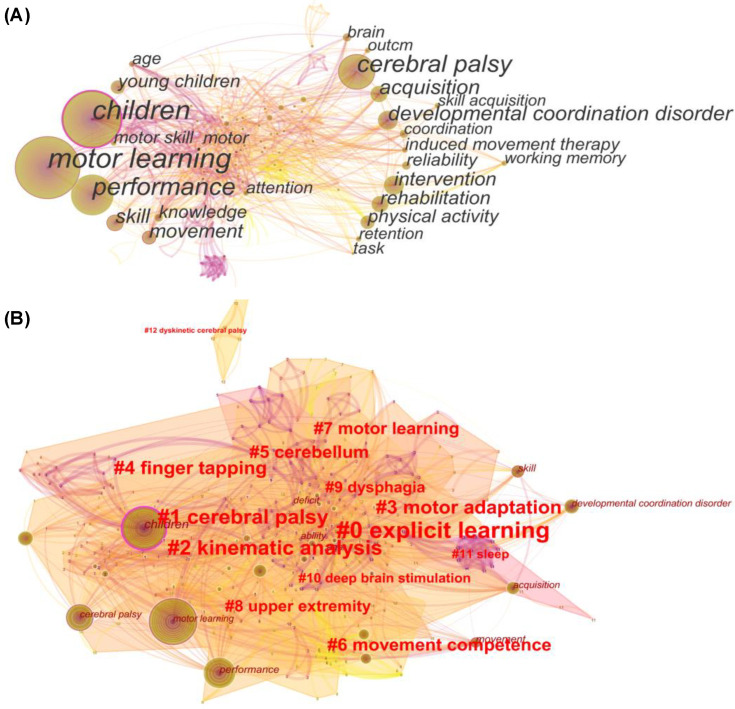
Keywords (**A**) and keyword clusters (**B**) in visualization.

Based on the approximate distribution, we presume the keywords can be divided into two interconnected categories (Figure 9A). The first category identifies characteristics of motor-learning research topics (primarily children, young children, skills, movement, and knowledge), while the second category identifies motor learning’s relationship to clinical symptoms, rehabilitation, and interventions. Furthermore, there are 13 relatively independent clusters of varying sizes (Figure 9B) that can and do correspond to the two keyword categories. It is worth noting that the cluster results frequently point to clinical symptoms (including #1 CP, #5 cerebellum, #9 dysphagia, #11 sleep, and #12 cognitive development) as well as diagnostic and intervention strategies (including #2 kinematic analysis, #motor adaptation, #4 finger tapping, #8 upper extremity, and #10 deep-brain stimulation) involving motor learning in preschoolers and children.

The strength and bursts of keywords in Table 2 represent the attention received and are used to forecast future research trends, with the beginning and ending years highlighted in red [24]. The strongest burst keyword was young children (5.26, burst in 2008), followed by reliability (4.93, 2018), memory (3.06, 2017), and instruction (2.91, 2017). Apart from their relevance to the keyword-clustering results, we found the keywords that emerged in 2016 and after 2016 to be especially intriguing. Furthermore, the application of new technological tools such as virtual reality (2017–2019) and tDCS (2020–2022) validity attracts attention.

## 4. Discussion

### 4.1. Quantity and Contribution Analysis

The longitudinal and transversal characteristics of motor learning in preschoolers and children were explored using bibliometric methods in this study. From 2006 to 2022, the number of publications increased gradually, with a clear upward trend. Overall, the United States (38.2%) and Australia (11.9%) have made important contributions, accounting for 50.1% (201/401) of the articles on this topic. The United States, Australia, Canada, England, the Netherlands, and Italy are not only at the forefront, but they also have relatively close cooperation and connections. Accordingly, in the last 15 years, renowned institutions and productive scholars in those countries have led the development. However, collaboration between institutions and scholars is much smaller in scale than collaboration between countries and has a distinct “regionalized” collaborative character (Figure 3B and Figure 4C).

### 4.2. Research Resources and Fundamental

Motor learning is a broad concept for a process that encompasses motor adaptation, skill acquisition, and decision-making and is dependent on the structural integrity and functional activity of the cortico-striatal circuit and the cerebellum [1]. The author and co-cited author’s information (Figure 4B,D and Figure 7), the research categories (Figure 5), and journal information (Figure 6) all combine to form a valuable research resource.

The names that stand out in motor learning and control are highlighted in the contribution and collaboration of the co-cited authors’ visualization. As we all know, many scholars have proposed and widely used theories in previous years. For example, Adams initially presented the closed-loop theory for simple motor learning [32]. Fitts and Posner, proposed the classical learning stage model in 1967, which included the cognitive, associative, and automation stages. Schmidt then proposed a definition of motor learning as well as a scheme theory [1,33]_ENREF_25, providing theoretical support for a wide range of fields and disciplines. In addition, professors Wulf, Novak, Wilson, Cohen, Oskoui, Gordon, Gheysen, Chiviacowsky, and others have conducted influential research. Even though those professors are not on the current list of highly published scholars, their research falls into important clusters, as evidenced by the top 10 co-cited references (Table 2). Thus, from this vantage point, their work is still making an important contribution to the development of motor learning in preschoolers and children.

The most important research categories for this topic, according to our findings, are Neurosciences and Neurology, Psychology, Rehabilitation, and Sport Sciences, which account for the majority of articles. The Education and Educational Research and Genetic and Heredity categories, in particular, have high centrality (Figure 5), indicating that significant research has emerged despite the low publication rates. Furthermore, co-cited references and co-cited journals are commonly regarded as the foundation for a specific research field, assisting knowledge-domain exploration. While the impact factors of journals ranked by publications and centrality varied (ranging from 1.33 to 6.56), most of the listed journals are flagships in the field of motor learning and hold significant positions in their categories. Professor Schmidt founded *J Motor Behav* and served as the first editor-in-chief. *Dev Med Child Neurol, J Motor Behav, and Res Q Exercise Sports* are among the top ten journals for both publications and centrality. Novak [18], Wilson [10], and Oskoui [28], all well-known researchers, all published critical articles in *Dev Med Child Neurol* the same year. Several high-centrality or high-publication journals in the categories of Neuroscience (*J Neurosci, Neuroimage*, *Neuropsychology*), Rehabilitation (*Clin Rehabil*), and Psychology (*Child Dev*, *J Exp Child Psychol*) deserve special attention in the field of motor learning in preschoolers and children. Getting published in these prestigious journals is still difficult today. Notably, Schmidt and Lee’s *Motor Control and Learning: A Behavioral Emphasis* and the American Psychiatric Association’s *Diagnostic Statistics Manuals* were influential and have become important works and manuals in the field.

### 4.3. Research Topic

Typically, the co-citation network is used to analyze valuable information about a specific intellectual link. In the last 15 years, we found that co-cited references clusters were primarily focused on clinical symptoms and pathogenesis indicators (Figure 7). According to the current results, CP (#0), DCD (#4), and motor skills disorder (#9) have risen to prominence. These diseases are extremely concerning because they are the leading causes of physical disability in preschoolers and have a long-term impact [34]. In fact, both CP and DCD cause mild to severe movement impairment. CP with mild/moderate motor impairments and motor-skills disorder, consistent with DCD, affects nearly half of all preterm babies and includes difficulties with balance, manual dexterity, and ball skills [17]. In addition, because DCD is more common in premature infants, follow-up should last at least 5 years. As a result, it is critical to understand and distinguish between the various motor impairments experienced by children, including mild motor impairment from CP and DCD, so that optimal interventions can be implemented early and caregivers understand long-term motor function.

Furthermore, clinical research and education-based interventions have focused on language impairment in preschoolers and children. As a result, speech disorders (#6), explicit learning (#2), and instructor-control feedback (#7) were all identified as important clusters. The primary cause of speech disorders (#6) is amyotrophic lateral sclerosis. Since the nervous system that controls voluntary muscles is damaged, motor nerves and facial muscles drop dead, making speaking and swallowing difficult. As a result, muscle coordination and facial muscle strength may influence children’s speech function. Besides, instructor-control feedback (#7) is equivalent to self-control feedback in improving acquisition and motor learning in CP children [15].

Previous research used motor learning as a critical theoretical foundation for intervention in CP children [18,29]. It has been established that bimanual training and constraint-induced movement therapy (CIMT) are beneficial for the rehabilitation of CP children [29]. According to Novak et al.’s system review, fitness training, bimanual training, and CIMT interventions were effective at both the body-structure and -function levels [18]. Therefore, studying the mechanisms and relationships between the brain and cognitive, emotional, and behavioral functions is inextricably linked to motor learning.

The inferior parietal lobe (#1) and hemispheric specialization (#5) have emerged as important clusters. The neural mechanisms involved in CP are varied but always involve an alteration to the developing brain, whereas the mechanisms for other motor impairments in children are not clear. The inferior parietal lobe (#1) is another focus topic of research, particularly among children with CP and DCD, due to its significant contribution to motor control. According to the prototypically hybrid view of motor control, damage to the inferior parietal lobe can result in control defects [10]. It has been demonstrated that in young children with parietal lobe damage, growth on the affected side is usually impaired. Similarly, a child with hemiparetic CP has less sensation and more difficulty with hand function than a child with only motor dysfunction. Impaired motor learning may be an important mechanism of brain damage. A smaller hand in a child with hemiparetic CP indicates involvement of the parietal lobe and vice versa. A child with reduced sensation in that limb has more difficulty with hand function than a child with solely motor dysfunction.

### 4.4. Frontiers and Progress

The evolution of keyword bursts and the end year could be used to deduce research frontiers [24]. However, only high-frequency-keywords data has the potential to ignore the idea of high-quality research. In this study, we combined burst keywords (Table 2) and keyword clusters (Figure 9B) with a timeline map of co-cited references (Figure 8). Overall, the frontiers and progress of motor learning in preschoolers and children can be categorized as follows.

- Progress of disease and behavioral characteristics. Deficit, CP, high functioning autism, abnormal, child apraxia of speech, dynamics, internal model, DCD, and strategy were hotspots from 2008 to 2013. Comprehensive updates on overall prevalence [28] and evidence-based effective interventions [18] for children with CP were investigated. Furthermore, performance deficits [10], attention [27], and movement therapy and motor-learning enhancement [29] during CP, DCD, and autism received special attention. Meanwhile, researchers focused on the cognition of gesture control and basic-motor-control impairments in children with autism and attention deficit hyperactivity disorder (ADHD) [11].
- Frontiers in brain mechanism research and diagnostic indicators advancement. From 2014 to 2016, the strongest keyword was “young children,” and brain mechanisms were identified as hotspots (burst keywords: brain, spectrum disorder, and language; keyword clusters: #5 cerebellum). Indeed, neuroplasticity during maturation, structural and functional change in response to motor learning, and motor development in health and disease are hotspots [3,35]. Cerebellar abnormalities are associated with abnormal motor learning, according to structured and quantified brain imaging [22]. A recent study discovered that during an autism spectrum disorder (ASD) model, peripheral somatosensory neurons could alter the brain’s inherently dynamic biological capacity because a gamma-aminobutyric acid type A receptor agonist (isoguvacine) from peripheral restriction decreased tactile sensitivity [36].
- Another frontier focuses on intervention and rehabilitation (burst keywords: intervention, plasticity, perception, language, skill acquisition, and external focus; keyword clusters: #2 kinematic analysis, #3 motor adaption, #4 finger tapping, and #6 movement competence). Children with CP and DCD have impaired visual perception, kinesthetic perception, cross-modal perception, and motor programming [5,10]. The interaction of the individual, task, and environment will influence motor-skill levels [5]. Typically, the skills are learned and refined through practice. Children may benefit from early intervention or rehabilitation to achieve optimal motor development. Furthermore, minor changes in task characteristics and context will assist in skill transfer [37]. Therefore, ecological approaches include school-based movement programs, and rehabilitation is a possibility. Notably, motor learning and the philosophy of family-centered practice are difficult to distinguish and determine which treatment approaches and treatment features are actually working [18].
- The use of new technologies in education and rehabilitation can be considered a frontier (from 2017 to 2022, burst keywords: memory, instruction, virtual reality, implicit, and individual and transcranial magnetic stimulation (TMS); keyword clusters: #10 deep brain stimulation; co-cited reference: #10 transcranial direct current stimulation (tDCS) and #16 cognitive processing). Three that stand out are virtual reality (VR), tDCS, and TMS. Evidence from a randomized controlled trial and a systematic review suggested that VR technology could improve motor skills in children with DCD [38] and CP [39], respectively. Aside from these, tDCS has been used in the rehabilitation of neurological and psychiatric disorders as a non-invasive stimulation technique [40]. It not only improves children’s motor-learning abilities, performance, and tolerance, but it also aids in the rehabilitation of children with cerebral palsy and developmental delay [41]. Furthermore, based on the evidence, TMS has potential benefits for fine-motor-skills rehabilitation in CP [42], induces foreign language learning within the primary motor cortex, and has behavioral benefits [43]. Overall, VR, tDCS, and TMS have the potential to be promising technological tools for children with motor deficits, even though the underlying brain mechanisms are largely unknown.
- Cognitive processes should not be overlooked in the preceding framework, which is thought to be linked to the mechanism of new technology. Feedback [15], attention [27], motor imagery [16], motivation [13], internal model building [44], and transfer [45] are all examples of cognitive processes. The question of how cognitive processing influences and determines motor learning remains intriguing and challenging. While cognitive-processing understanding progresses slowly, it will become more important in children’s motor learning.

### 4.5. Strengths and Limitations

To the best of our knowledge, this is the first bibliometric study that identifies and characterizes research on motor learning in preschoolers and children. We provide a set of clear visual analyses based on quantitative and contribution analyses of countries/regions, institutes, authors, research categories, and publications, as well as co-citations and clusters.

The current analysis had a few limitations. Although WoSCC contains enough high-quality journals to analyze the relevant and complete literature data, potential bias from excluding other databases (PubMed, Scopus, Medline, et al.) may affect our results. However, due to current limitations in software, only WoSCC data can be used in CiteSpace for citation analysis in international collaboration. Furthermore, even though we developed comprehensive and prudent search strategies and included only searched articles based strictly on titles and abstracts, there may still be overlooked articles [26]. All these issues should be addressed in future research.

## 5. Conclusions

We conducted a bibliometric and comparative analysis over the last 15 years to provide a broad overview of research topics and frontiers in motor learning for preschoolers and children. Our findings indicate that the number of studies is constantly increasing, and that the United States and Australia, as well as their productive institutions and authors, hold key and central positions in this important research area. The dominant disciplines were Neuroscience and Neurology, Psychology, Rehabilitation, and Sport Sciences. In this regard, the journals *Developmental Medicine & Child Neurology*, *Human Movement Science*, *Physical Therapy*, *Neuropsychology*, *Journal of Motor Behavior*, and *Journal of Experimental Child Psychology* have been the most productive and pivotal.

The research topics have been focused on clinical symptoms (cerebral palsy, developmental coordination disorder, and autism), language impairment (speech disorders, explicit learning, and instructor-control feedback), and effective interventions. The research frontiers and progress have shifted to brain mechanisms and diagnostic indicator advancements, as well as new intervention and rehabilitation technological tools (virtual reality, transcranial magnetic stimulation, and transcranial direct current stimulation). Moreover, the cognitive process is critical in intervention, rehabilitation, and the application of new technologies and should not be overlooked.

In response to this field’s research key topics and frontiers, our broad overview identifies three major areas: brain-mechanism research has been an important foundation; clinical practice (intervention and rehabilitation) is the main application area; and new-technology application represents the prospect and vision.

## Figures and Tables

**Table 1:** Top 10 co-cited references on motor learning in preschoolers and children.

Ranking	Frequency	Centrality	Source	Co-Cited Reference	The First Author (Year)	Cluster
1	33	0.11	*Dev Med Child Neurol*	A systematic review of interventions for children with cerebral palsy: state of the evidence [18]	Novak (2013)	#0 cerebral palsy
2	17	0.13	Champaign, IL: Human Kinetics	Motor control and learning: a behavioral emphasis (4th ed.) * [1]	Schmidt (2005)	#5 hemispheric specialization
3	16	0.06	Diagnostic Stat Manu	American Psychiatric Association Frequency of citations * [6]	APA *	#4 DCD
4	15	0.03	*Int Rev Sport Exer Psych*	Attentional focus and motor learning: a review of 15 years [27]	Wulf (2013)	#2 explicit learning
5	13	0.01	*Psychon Bull Rev*	Optimizing performance through intrinsic motivation and attention for learning: The OPTIMAL theory of motor learning [13]	Wulf (2016)	#2 explicit learning
6	11	0.11	*Dev Med Child Neurol*	Understanding performance deficits in developmental coordination disorder: a meta-analysis of recent research [10]	Wilson (2013)	#4 DCD
7	8	0.04	*Dev Med Child Neurol*	An update on the prevalence of cerebral palsy: a systematic review and meta-analysis [28]	Oskoui (2013)	#0 cerebral palsy
8	8	0.03	*Neurorehabil Neural Repair*	Bimanual training and constraint induced movement therapy in children with hemiplegic cerebral palsy: a randomized trial [29]	Gordon (2011)	#3 splints
9	8	0.03	*Res Dev Disabil*	Impaired visuo-motor sequence learning in developmental coordination disorder [30]	Gheysen (2011)	#4 DCD
10	7	0.02	*J Intellect Disabil Res*	An external focus of attention enhances motor learning in children with intellectual disabilities [31]	Chiviacowsky (2013)	#2 explicit learning

Note: * indicates a book.

**Table 2:** Top 25 keywords with the strongest citation bursts.

Keywords	Strength	Begin	End	Years (2006–2022)
deficit	2.82	2008	2014	▂▂▃▃▃▃▃▃▃▂▂▂▂▂▂▂▂
high functioning autism	2.31	2008	2012	▂▂▃▃▃▃▃▂▂▂▂▂▂▂▂▂▂
abnormality	2.30	2008	2013	▂▂▃▃▃▃▃▃▂▂▂▂▂▂▂▂▂
motor	2.43	2010	2015	▂▂▂▂▃▃▃▃▃▃▂▂▂▂▂▂▂
childhood apraxia of speech	2.28	2010	2012	▂▂▂▂▃▃▃▂▂▂▂▂▂▂▂▂▂
dynamics	2.80	2011	2012	▂▂▂▂▂▃▃▂▂▂▂▂▂▂▂▂▂
internal model	2.29	2012	2013	▂▂▂▂▂▂▃▃▂▂▂▂▂▂▂▂▂
developmental coordination disorder	2.53	2013	2014	▂▂▂▂▂▂▂▃▃▂▂▂▂▂▂▂▂
strategy	2.10	2013	2017	▂▂▂▂▂▂▂▃▃▃▃▃▂▂▂▂▂
young children	5.26	2014	2016	▂▂▂▂▂▂▂▂▃▃▃▂▂▂▂▂▂
intervention	2.56	2014	2017	▂▂▂▂▂▂▂▂▃▃▃▃▂▂▂▂▂
brain	2.72	2015	2017	▂▂▂▂▂▂▂▂▃▃▃▂▂▂▂▂▂
spectrum disorder	2.07	2015	2016	▂▂▂▂▂▂▂▂▂▃▃▃▂▂▂▂▂
plasticity	2.89	2016	2017	▂▂▂▂▂▂▂▂▂▃▃▂▂▂▂▂▂
perception	2.37	2016	2018	▂▂▂▂▂▂▂▂▂▂▃▃▃▂▂▂▂
language	2.09	2016	2018	▂▂▂▂▂▂▂▂▂▂▃▃▃▂▂▂▂
skill acquisition	2.08	2016	2017	▂▂▂▂▂▂▂▂▂▂▃▃▂▂▂▂▂
external focus	2.07	2016	2019	▂▂▂▂▂▂▂▂▂▂▃▃▃▃▂▂▂
memory	3.06	2017	2019	▂▂▂▂▂▂▂▂▂▂▂▃▃▃▂▂▂
instruction	2.91	2017	2018	▂▂▂▂▂▂▂▂▂▂▂▃▃▂▂▂▂
virtual reality	2.05	2017	2019	▂▂▂▂▂▂▂▂▂▂▂▃▃▃▂▂▂
reliability	4.93	2018	2019	▂▂▂▂▂▂▂▂▂▂▂▂▃▃▂▂▂
implicit	2.42	2018	2019	▂▂▂▂▂▂▂▂▂▂▂▂▃▃▂▂▂
individual	2.38	2018	2019	▂▂▂▂▂▂▂▂▂▂▂▂▃▃▂▂▂
transcranial magnetic stimulation	2.40	2020	2022	▂▂▂▂▂▂▂▂▂▂▂▂▂▂▃▃▃

Note: Begin, start time of keyword burst; End, end time of keyword burst. Legend: ▂ one year; ▃ burst year.

## Data Availability

The data presented in this study are available in this manuscript.

## Supplementary Materials

The following supporting information can be downloaded at: https://www.mdpi.com/article/10.3390/healthcare10081415/s1.

## Appendix A

**Table A1:** Summary and description of the top 10 clusters.

Cluster ID	Size	Silhouette	Label (LLR)	Mean (Cite Year)	Description
#0	48	0.925	cerebral palsy	2012	A group of disorders that affect movement, muscle tone, balance, and posture.
#1	47	0.975	inferior parietal lob	2006	The inferior parietal lobe (IPL) is a key neural matrix that determines basic attention, language, and social cognition in human interaction.
#2	46	0.955	explicit learning	2014	Learning that results from clearly stated directions or instructions.
#3	43	0.971	splints	2005	A rigid or flexible device used to fix a displaced or movable part.
#4	39	0.956	developmental coordination disorder	2011	Exceptional clumsiness, or an unusual delay in meeting motor milestones of childhood, when such a delay results in functional impairment and cannot be attributed to other medical conditions.
#5	38	0.908	hemispheric specialization	2006	The control of distinct neurological functions in the right and left hemispheres of the brain.
#6	29	0.987	speech disorders	2010	A language disorder is an impairment in comprehension use of a spoken, written, or other symbol system.
#7	24	0.885	instructor-control feedback	2012	Feedback from others.
#9	19	0.896	motor skills disorder	2013	Symptoms of motor-skill learning difficulties.
#10	19	1.000	transcranial direct current stimulation	2015	A popular brain stimulation method that is used to modulate cortical excitability, producing facilitatory or inhibitory effects upon a variety of behaviors.

Note: Size indicates the number of references that a cluster contains; Silhouette means the homogeneity of a cluster. (S > 0.5, the cluster is reasonable; S > 0.7, the cluster is convincing; S < 0.5, the cluster will be lost); LLR, log likelihood ratio.
