# Supplementary material for: A Bibliometric and Visualization Analysis of Motor Learning in Preschoolers and Children over the Last 15 Years
Source: Healthcare (Basel). 2022 Jul 28;10(8):1415. doi: 10.3390/healthcare10081415 (PMC9407894; doi:10.3390/healthcare10081415)
Supplement: Supplementary file 1 [file healthcare-10-01415-s001.zip › healthcare-1807720-supplementary.pdf]

Supplementary S1. Specific search process table.

Set	Result	Topic
#20	418	#15 NOT #19
#19	1,450,474	#16 OR #17 OR #18
#18	652,442	TOPIC: ("rat ") Indexes=SCI-EXPANDED, SSCI, A&HCI, ESCI, CCR-EXPANDED, IC. Document Types= Article OR Review. Language =English. Timespan =01/01/1975-
#17	935,699	TOPIC: ("mice ") Indexes=SCI-EXPANDED, SSCI, A&HCI, ESCI, CCR-EXPANDED, IC. Document Types= Article OR Review. Language =English. Timespan =01/01/1975-
#16	935,712	TOPIC: ("mouse") Indexes=SCI-EXPANDED, SSCI, A&HCI, ESCI, CCR-EXPANDED, IC. Document Types= Article OR Review. Language =English. Timespan =01/01/1975-
#15	499	#13 AND #14
#14	5,864	TOPIC: ("motor learning") Indexes=SCI-EXPANDED, SSCI, A&HCI, ESCI, CCR-EXPANDED, IC. Document Types= Article OR Review. Language =English. Timespan =01/01/1975-
#13	1,025,072	#8 NOT #12
#12	1,339,806	#9 OR #10 OR #11
#11	9,936	TOPIC: ("teenager") Indexes=SCI-EXPANDED, SSCI, A&HCI, ESCI, CCR-EXPANDED, IC. Document Types= Article OR Review. Language =English. Timespan =01/01/1975-
#10	300,214	TOPIC: ("adolescent") Indexes=SCI-EXPANDED, SSCI, A&HCI, ESCI, CCR-EXPANDED, IC. Document Types= Article OR Review. Language =English. Timespan =01/01/1975-
#9	1,115,877	TOPIC: ("adult") Indexes=SCI-EXPANDED, SSCI, A&HCI, ESCI, CCR-EXPANDED, IC. Document Types= Article OR Review. Language =English. Timespan =01/01/1975-
#8	1,396,888	#1 OR #2 OR #3 OR #4 OR #5 OR #6 OR #7 OR #8
#7	177,633	TOPIC: ("young child*") Indexes=SCI-EXPANDED, SSCI, A&HCI, ESCI, CCR-EXPANDED, IC. Document Types= Article OR Review. Language =English. Timespan =01/01/1975-
#6	182,037	TOPIC: ("early child*") Indexes=SCI-EXPANDED, SSCI, A&HCI, ESCI, CCR-EXPANDED, IC. Document Types= Article OR Review. Language =English. Timespan =01/01/1975-
#5	10,473	TOPIC: ("kindergarten") Indexes=SCI-EXPANDED, SSCI, A&HCI, ESCI, CCR-EXPANDED, IC. Document Types= Article OR Review. Language =English. Timespan =01/01/1975-
#4	22,488	TOPIC: ("pupil") Indexes=SCI-EXPANDED, SSCI, A&HCI, ESCI, CCR-EXPANDED, IC. Document Types= Article OR Review. Language =English. Timespan =01/01/1975-
#3	313,573	TOPIC: ("kid*") Indexes=SCI-EXPANDED, SSCI, A&HCI, ESCI, CCR-EXPANDED, IC. Document Types= Article OR Review. Language =English. Timespan =01/01/1975-
#2	41,896	TOPIC: ("preschool*") Indexes=SCI-EXPANDED, SSCI, A&HCI, ESCI, CCR-EXPANDED, IC. Document Types= Article OR Review. Language =English. Timespan =01/01/1975-
#1	1,087,823	TOPIC: ("child*") Indexes=SCI-EXPANDED, SSCI, A&HCI, ESCI, CCR-EXPANDED, IC. Document Types= Article OR Review. Language =English. Timespan =01/01/1975-

Table of the Specific search process. Note: Indexes=SCI-EXPANDED, SSCI, A&HCI, ESCI, CCR-EXPANDED, IC. Document Types= Article OR Review. Language =English. Timespan =01/01/1975-
